# Errata

**Published:** 1888-04

**Authors:** 


ERRATA.—Though we do our best to avoid them, errors will creep into our issues. In our February number, the name of Dr. George H. Clark was inadvertently omitted from the Committee signing the Memorial Resolutions of the Hahnemannian Association of Pennsylvania. In the article of Dr. M. W. Van Denburg, the following errata are to be noted: Page 78, line 2, for "*our point*," read "*one point;*" line 13, for "*rich*," read "*sick;*" line 25, for "*brain*," read "*pain;*" third line from bottom, for "*his meditative nature*," read "*vis medicatrix naturæ*."

In the *March* number, page 142, twentieth line from bottom, for *central*, read *cerebral*. Page 156, second line, for *evening*, read *morning;* seventh line, for *Spongia*, read *Spigelia*.

